# Bacterial Butyrate in Parkinson's Disease Is Linked to Epigenetic Changes and Depressive Symptoms

**DOI:** 10.1002/mds.29128

**Published:** 2022-06-20

**Authors:** Aoji Xie, Elizabeth Ensink, Peipei Li, Juozas Gordevičius, Lee L. Marshall, Sonia George, John Andrew Pospisilik, Velma T.E. Aho, Madelyn C. Houser, Pedro A.B. Pereira, Knut Rudi, Lars Paulin, Malú G. Tansey, Petri Auvinen, Patrik Brundin, Lena Brundin, Viviane Labrie, Filip Scheperjans

**Affiliations:** ^1^ Department for Neurodegenerative Science, Parkinson's Disease Center Van Andel Institute Grand Rapids Michigan USA; ^2^ Center for Epigenetics Van Andel Institute Grand Rapids Michigan USA; ^3^ Department of Neurology Helsinki University Hospital, and Clinicum, University of Helsinki Helsinki Finland; ^4^ Institute of Biotechnology, DNA Sequencing and Genomics Laboratory University of Helsinki Helsinki Finland; ^5^ Nell Hodgson Woodruff School of Nursing Emory University Atlanta Georgia USA; ^6^ Department of Physiology Emory University School of Medicine Atlanta Georgia USA; ^7^ Faculty of Chemistry, Biotechnology and Food Science (KBM) Norwegian University of Life Sciences Ås Norway; ^8^ Department of Neuroscience and Neurology, Center for Translational Research in Neurodegenerative Disease University of Florida College of Medicine Gainesville Florida USA; ^9^ Division of Psychiatry and Behavioral Medicine, College of Human Medicine Michigan State University Grand Rapids Michigan USA

**Keywords:** Parkinson's disease, DNA methylation, microbiome, epigenetics, gut brain axis

## Abstract

**Background:**

The gut microbiome and its metabolites can impact brain health and are altered in Parkinson's disease (PD) patients. It has been recently demonstrated that PD patients have reduced fecal levels of the potent epigenetic modulator butyrate and its bacterial producers.

**Objectives:**

Here, we investigate whether the changes in the gut microbiome and associated metabolites are related to PD symptoms and epigenetic markers in leucocytes and neurons.

**Methods:**

Stool, whole blood samples, and clinical data were collected from 55 PD patients and 55 controls. We performed DNA methylation analysis on whole blood samples and analyzed the results in relation to fecal short‐chain fatty acid concentrations and microbiota composition. In another cohort, prefrontal cortex neurons were isolated from control and PD brains. We identified genome‐wide DNA methylation by targeted bisulfite sequencing.

**Results:**

We show that lower fecal butyrate and reduced counts of genera *Roseburia*, *Romboutsia,* and *Prevotella* are related to depressive symptoms in PD patients. Genes containing butyrate‐associated methylation sites include PD risk genes and significantly overlap with sites epigenetically altered in PD blood leucocytes, predominantly neutrophils, and in brain neurons, relative to controls. Moreover, butyrate‐associated methylated‐DNA regions in PD overlap with those altered in gastrointestinal (GI), autoimmune, and psychiatric diseases.

**Conclusions:**

Decreased levels of bacterially produced butyrate are related to epigenetic changes in leucocytes and neurons from PD patients and to the severity of their depressive symptoms. PD shares common butyrate‐dependent epigenetic changes with certain GI and psychiatric disorders, which could be relevant for their epidemiological relation. © 2022 The Authors. *Movement Disorders* published by Wiley Periodicals LLC on behalf of International Parkinson and Movement Disorder Society

Parkinson's disease (PD) is a neurodegenerative disorder that is typically characterized by motor impairments due to the death of dopaminergic neurons located within the substantia nigra pars compacta.^1^ A hallmark of PD is the aggregation of misfolded α‐synuclein protein in both the central and peripheral nervous system, forming aggregates or Lewy bodies.^2^ The discovery of α‐synuclein aggregates in the enteric nervous system, coupled with the early gastrointestinal (GI) symptoms of PD (eg, constipation), has led to the hypothesis that the pathogenesis of PD may originate in the GI tract or at least outside of the central nervous system (CNS).^3^ In addition, it is hypothesized that α‐synuclein aggregates can be transported in a prion‐like manner from the enteric nervous system to the CNS through the vagus nerve.^4^ This hypothesis is supported by the decreased risk for PD among patients who have undergone a vagotomy.^5^ Further, intertwining the GI system with PD is the finding that the presence of the gut microbiome is required for mice that overexpress α‐synuclein to develop motor deficits.^6^ These same mice displayed increased motor deficits after receiving a fecal transplant from a PD patient compared to receiving transplant from a healthy donor. Moreover, the composition and function of the gut microbiome in PD patients have been shown to be significantly different from healthy controls in multiple studies.^7^, ^8^, ^9^, ^10^

Recent meta‐analyses suggest that a reduced abundance of short‐chain fatty acid (SCFA) producing bacteria is one of the most consistent findings in the PD microbiome composition across studies.^11^, ^12^ SCFAs are saturated fatty acids produced via the fermentation of dietary fiber by certain colonic bacteria.^13^ The deficiency of SCFAs has been implicated in multiple diseases such as autoimmune disorders, cancer, metabolic syndromes, and neurological disorders.^14^ Fecal samples from PD patients have been shown to harbor significantly lower concentrations of the SCFAs acetate, propionate, and butyrate when compared to healthy controls.^15^ Beyond its central role as the main energy source for colonocytes, butyrate, specifically, has been implicated as an important bacterial metabolite due to its role as a strong endogenous histone deacetylase (HDAC) inhibitor indirectly affecting DNA methylation,^16^ allowing it to epigenetically alter the gene expression of multiple cell types.^17^ Butyrate is also the ligand of some free fatty acid receptors that are critical for inflammation regulation and secretion of peptide hormones.^18^ Several studies have demonstrated the ability of butyrate to reduce the inflammatory properties of both innate and adaptive immune cells through inhibiting reactive oxygen species release and inflammatory cytokine production and inducing activated immune cell apoptotic mechanisms.^19^, ^20^, ^21^ In addition to its immune‐modulating properties, butyrate has been shown in multiple in vivo studies to influence the CNS through decreasing blood–brain barrier permeability, decreasing microglial activation, and relieving anxiety and depression, which are both common prodromal PD symptoms.^22^, ^23^, ^24^, ^25^ Butyrate has also shown its impact on astrocyte gene expression in vitro and neuroprotective effects in PD mouse models.^26^, ^27^, ^28^ Some studies also suggested deleterious effects of SCFAs.^6^, ^29^, ^30^ However, the possible impact of the altered butyrate level observed in PD patients on epigenome status as well as on clinical symptoms has yet to be elucidated.

To test whether butyrate impacts epigenetic markers in the blood and brain of PD patients, and whether this is associated with symptom severity, we performed DNA methylation profiling in whole blood samples and neuronal tissue from two cohorts of PD patients and controls (Fig. 1) and related the findings to fecal gut microbiome and metabolite data and clinical symptoms.

**FIG 1:**
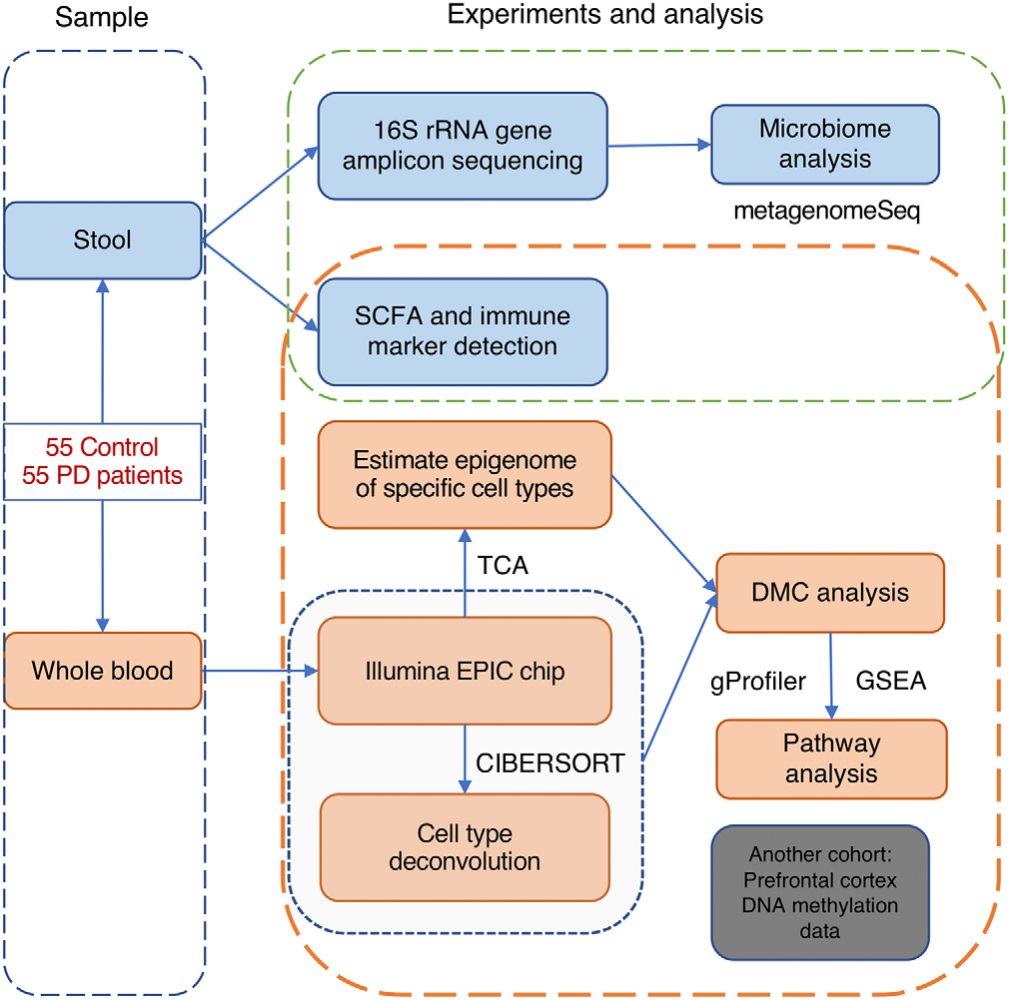
Outline of this study. [Color figure can be viewed at wileyonlinelibrary.com]

## Patients and Methods

### Human Samples and Metadata

Blood samples, clinical data, microbiome count data, inflammatory and permeability markers, and stool SCFA levels used in this study are from the Helsinki Parkinson microbiome cohort, and methodology of sampling, sample processing, and analysis have been described previously.^31^, ^32^ The study was approved by the ethics committee of the Hospital District of Helsinki and Uusimaa. All participants provided informed consent. Human prefrontal cortex tissue for this study was obtained from the Parkinson's UK Brain Bank, NIH NeuroBioBank, and Michigan Brain Bank, with approval from the ethics committee of the Van Andel Research Institute (IRB 15025). Methodology of sampling, sample processing, and analysis have been described previously.^33^ We used the differential methylated cytosine data (Supplementary File 5) in our analysis.

### Statistical Analyses

We performed statistical analyses with R (v3.6.1),^34^ with packages such as metagenomeSeq (v 1.27.3)^35^ for differential microbial data comparisons, ChAMP (v 2.20.1)^36^ and Minfi (v 1.31.1)^37^ for EPIC array analysis, tensor composition analysis (TCA) (v 1.1.0)^38^ for epigenome estimation of immune cell types, limma (v 3.41.17)^39^ for robust linear regression, and ggplot2 (v 3.3.1)^40^ for data visualization. All the codes used for this work are publicly available: Github: https://github.com/AojiXie/PD_microbiome_DNA_methylation.

### Genome‐Wide DNA Methylation Profiling

Whole‐genome DNA methylation profiling for each sample was performed on Illumina Methylation EPIC BeadChip microarrays at Van Andel Institute Genomic core. Bisulfite‐converted DNA samples (n = 136, including replicates) were randomized across arrays (eight samples per array). Data generated from the microarrays were preprocessed using Minfi (v 1.31.1).^37^ Normalization was performed using Noob.^41^ We confirmed that the sex of the individuals matched that inferred from the DNA methylome (minfi getSex() function).

The portions of immune cell types (CD8^+^T cell, CD4^+^T cell, B cell, NK, monocyte, and neutrophil) were estimated by CIBERSORT^42^ (Fig. S2a) using whole blood‐specific markers as reference.^43^ The filtering method in ChAMP was used to filter probes: probes that overlapped single nucleotide polymorphisms (SNPs)^44^ (minor allele frequency >0.05) on the CpG or single‐base extension (95,485 probes), probes that aligned to multiple locations (42,558 probes), probes with a beadcount <3 in at least 5% of samples (3380 probes), MultiHit Start^45^ (11 probes), probes located on X,Y chromosome (16,541 probes), NoCG Start (2953), and those that failed detectability (*P* > 0.01) (7951 probes) were excluded. After processing, 739,597 probes remained. Champ.svd() in ChAMP was used to test the batch effects. Those batch effects (array and slide positions) were corrected by ComBat.^46^ After batch effects correction, the M value was ready for the subsequent statistical analysis. Cell‐type‐specific resolution epigenetics were performed using TCA.^38^

### Statistical Analysis for Differentially Methylated Sites

DNA methylation analysis involved robust linear regression models with empirical Bayes from the *limma* (v 3.41.17) statistical package.^39^ *P*‐values were adjusted with a Benjamini–Hochberg correction for multiple testing, and those with false discovery rate (FDR) *q* < 0.05 were deemed significant.

### Model

## 1: In Whole Blood Methylation Epigenome

Variable selection: cell‐type percentages were used as covariates because the whole blood contains several immune cell types in varying proportions. This variation might affect the interpretations of DNA methylation levels based on whole blood DNA.^47^ Body mass index (BMI) and smoking history are used as covariates because their associations with whole blood DNA methylation have been observed.^48^, ^49^

M value ~ butyrate + age + sex + smoking history + BMI + CD4^+^T cell + CD8^+^T cell + B cell + monocyte + neutrophil.

## 2: In Cell‐Specific Epigenome

M value ~ butyrate + age + sex + smoking history +  BMI.

### Pathway Enrichment Analysis

To identify proximal interactions with gene targets, we used the *GREAT* (v4.0.4) software.^50^ Gene annotation was performed for the gene targets of the significant cytosine sites in our analysis and for the background, consisting of gene targets for all cytosines included in our analysis. The background consisted of 18,455 genes. Pathway analysis of methylated cytosines altered in PD and correlated with butyrate level was performed using g:Profiler,^51^ with networks determined by EnrichmentMap and clustered by AutoAnnotate in *Cytoscape* (v3.7.1).^52^

Because enhancer elements dynamically regulate gene expression through three‐dimensional physical interactions, we analyzed chromatin interaction data to reveal the gene targets of enhancers relevant to the differential methylation sites linked to butyrate. For this analysis, we used promoter‐centric chromatin interactions identified in blood cell types.^53^ Gene enrichment set test of different blood cell types was performed by GSEA^54^, ^55^ (https://www.gsea-msigdb.org/gsea/msigdb/index.jsp) Reactome gene sets. Benjamini–Hochberg FDR *q* < 0.01 with minimal gene set less than 100 was used as the significant threshold.

### Genetic–Epigenetic Correlation

Genetic–epigenetic analyses were performed using *LD Score* software^56^, ^57^ to estimate the correlations between butyrate‐associated methylated‐DNA (mDNA) regions and the genome‐wide association studies (GWAS) summary statistics of other diseases. To construct butyrate‐associated mDNA regions for linkage disequilibrium (LD) score regression analysis, SNPs within ±5000 bp of EPIC chip array sites were included, and the *P*‐values of methylation cytosines in butyrate linear model were assigned to those SNPs. If an SNP was within ±5000 bp of more than one methylated cytosine, the smallest *P*‐value was selected. The summary statistic of a 2019 PD GWAS study^58^ was used in this analysis. For other diseases, we used the summary statistics that are provided in the LD Hub interface.^59^ *P* < 0.05 was used as the significance threshold.

The common SNPs (with *P* < 0.05) between butyrate‐associated mDNA regions, PD GWAS summary statistics, and other diseases were extracted. *GREAT* ^50^ was used to obtain the gene annotation of those common SNPs with association rule: basal + extension: 5000 bp upstream, 1000 bp downstream, 600,000 bp maximum extension, curated regulatory domains included. Gene set enrichment analysis was performed by GSEA using Reactome gene sets^54^, ^55^ (https://www.gsea-msigdb.org/gsea/msigdb/index.jsp). Benjamini–Hochberg FDR *q* < 0.01 with minimal gene set less than 100 was used as the significant threshold.

### Data Availability

Microbiota data are available at the European Nucleotide Archive (accession number: PRJEB27564) (https://www.ebi.ac.uk/ena/browser/view/PRJEB27564). Other data and files utilized in this study are available from the corresponding authors on reasonable request.

## Results

### Butyrate‐Producing Microbes Are Altered in PD and Correlate with Depressive Symptoms

**FIG 2:**
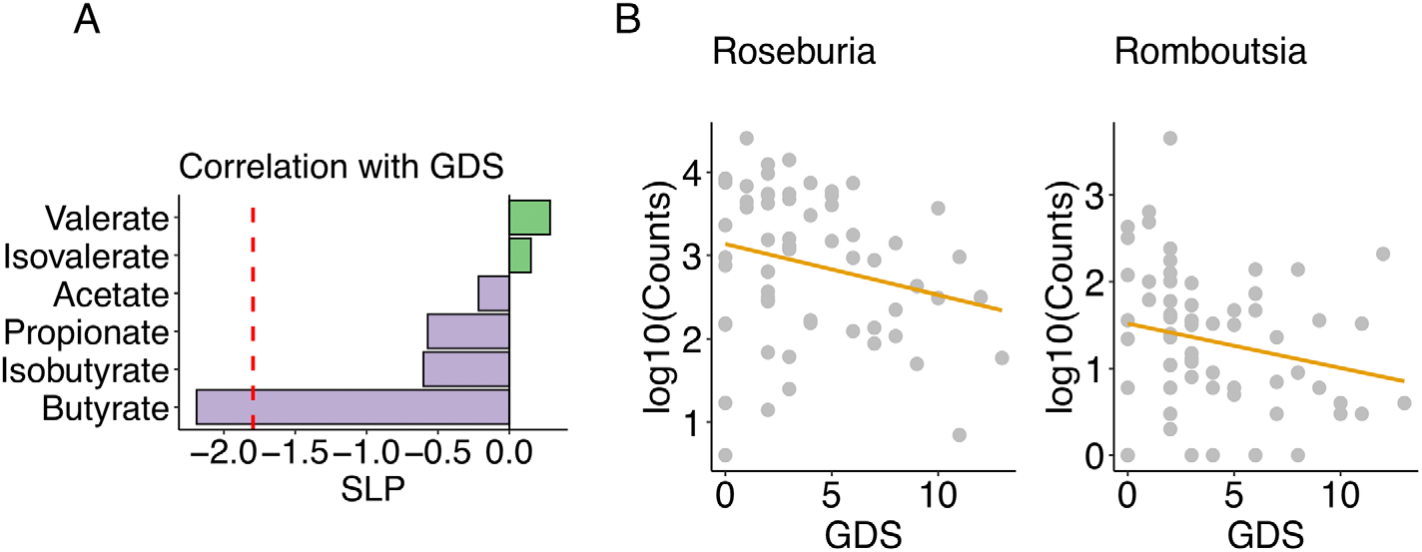
Butyrate and butyrate‐producing microbes are associated with PD depressive symptoms. (**A**) Short‐chain fatty acids change with Geriatric Depression Scale (GDS) total scores in PD patients (robust linear regression, adjusting for age, sex, smoking status, and BMI [body mass index]. Benjamini–Hochberg FDR [false discovery rate] *q* < 0.05 is used as the significant threshold). (**B**) The butyrate‐producing bacterial genera *Roseburia* and *Romboutsia* were negatively associated with GDS (Geriatric Depression Scale) total scores in PD patients. Benjamini–Hochberg FDR (false discovery rate) *q* < 0.05, signed logP: SLP. [Color figure can be viewed at wileyonlinelibrary.com]

First, we reanalyzed previously published^31^, ^32^ raw data on SCFA levels and 16S rRNA gene amplicon counts from stool samples of PD patients and healthy controls. The metagenomeSeq was used for differential microbial analysis.^35^ Unlike RNAseq studies, most operational taxonomic units are rare (absent from a large number of samples) because of insufficient sequencing depth (undersampling) or some organisms being present only in a few samples. This sparsity can lead to strong biases when sequence read counts are tested for significant differences. Zero counts in samples with low coverage are misinterpreted as absent taxonomic features. The advantages of metagenomeSeq are the zero‐inflated Gaussian (ZIG) mixture model that removes testing biases resulting from undersampling and the cumulative‐sum scaling normalization method to avoid biases of uneven sequencing depth.^60^ We can confirm significantly reduced butyrate levels (Benjamini–Hochberg FDR *q* < 0.05, robust linear regression) (Fig. APPENDIX S1a) and differential abundances of the genera *Bifidobacterium, Butyricicoccus, Clostridium_XlVa, Lactobacillus, Prevotella*, and *Roseburia* in PD patients (Benjamini–Hochberg FDR *q* < 0.05, metagenomeSeq ZIG model) (Fig. APPENDIX S1b).^31^ Important new findings within the PD group are links between depressive symptoms as measured using the Geriatric Depression Scale (GDS‐15) and lower fecal butyrate levels (Benjamini–Hochberg FDR *q* < 0.05, robust linear regression) (Fig. 2A) as well as lower counts of the genera *Prevotella, Romboutsia*, and *Roseburia* and higher counts of *the genera Deltaproteobacteria_unclassified* (Benjamini–Hochberg FDR *q* < 0.05, metagenomeSeq ZIG model) (Fig. APPENDIX S1c; Fig. 2B). In line with these findings, we confirm a positive correlation of the genera *Romboutsia* and *Roseburia* with butyrate levels in PD patients (Fig. APPENDIX S1c).^32^ In addition, no other symptoms, including GI (Rome‐III questionnaire; Wexner score), motor symptoms (the Unified Parkinson's Disease Rating Scale [UPDRS]), and nonmotor symptoms (Non‐Motor Symptoms Questionnaire [NMSQ] and Non‐Motor Symptoms Scale [NMSS]), were linked with butyrate in PD patients.

### Fecal Butyrate Levels Are Associated with Epigenetic Alterations in Leucocytes and Neurons of PD Patients

**FIG 3:**
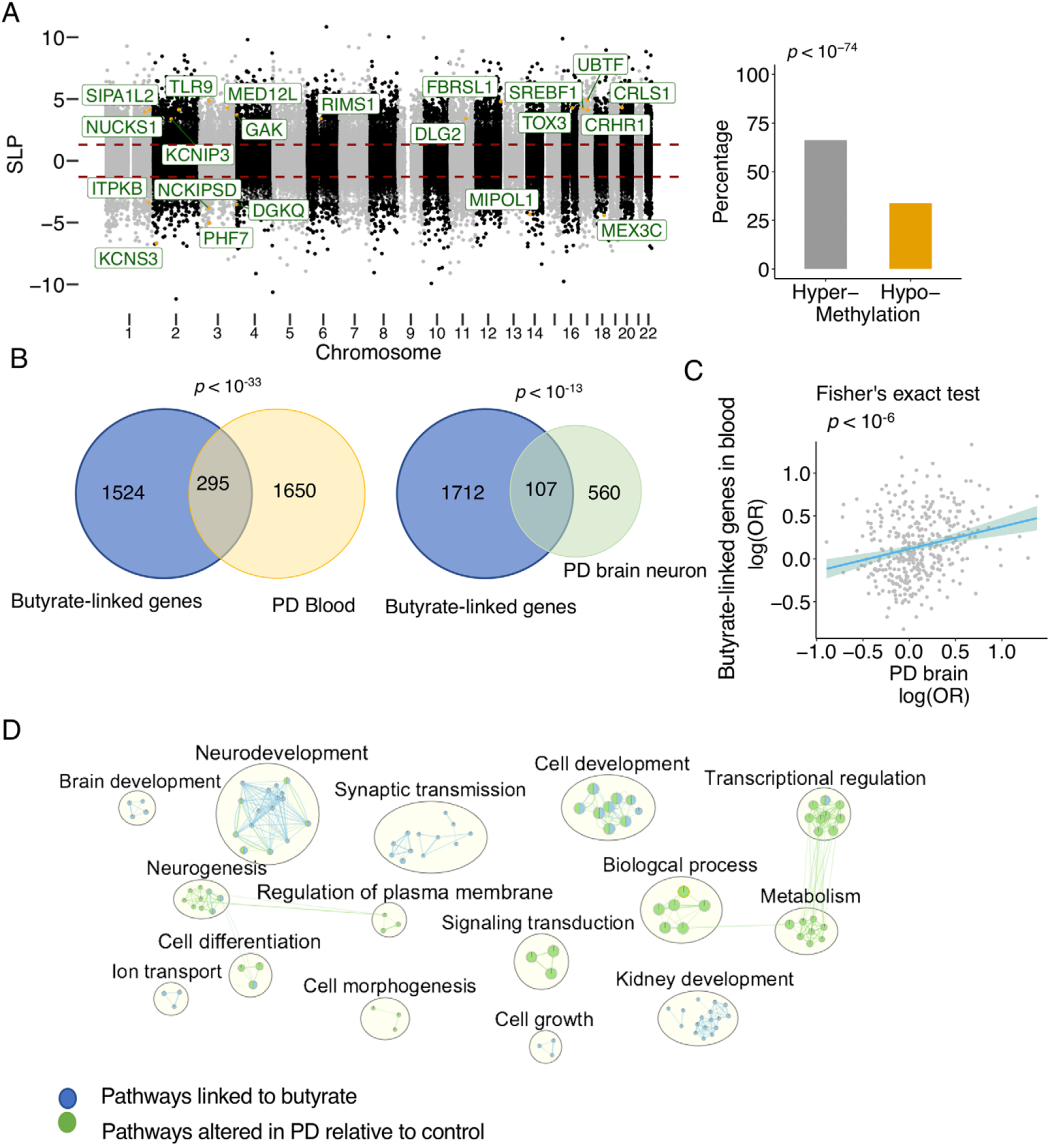
Links of butyrate to PD epigenome in brain and blood. DNA methylation analysis was performed by Illumina EPIC array in PD and control whole blood (n = 55 PD, 55 controls). (**A**) DNA methylation changes in the blood of PD patients associated with butyrate levels are identified (robust linear regression, 3195 cytosines at Benjamini–Hochberg FDR [false discovery rate] *q* < 0.05, after adjusting for age, sex, smoking status, BMI [body mass index], and blood cell types). PD risk genes (identified by GWAS [genome‐wide association studies]) with differential methylation are highlighted. The percentage of hypermethylated and hypomethylated cytosines is plotted (Fisher's test, *P* < 0.05), and butyrate level is positively associated with cytosine methylation in PD. (**B**) Genes in PD blood (295 genes) and PD prefrontal cortex (107 genes) are significantly converged on the genes linked to butyrate levels (Fisher's exact test, *P* < 0.05). (**C**) Epigenetic alterations in blood and brain converge on those methylation sites linked to butyrate. The log(odds ratio) of genes in PD blood is significantly correlated with the log(odds ratio) of those genes in PD prefrontal cortex (Fisher's exact test, *P* < 0.05). (**D**) Enrichment analysis of genes epigenetically linked to butyrate and altered in PD relative to control. Nodes are *q* < 0.05 pathways merged by EnrichmentMap in Cytoscape. [Color figure can be viewed at wileyonlinelibrary.com]

We identified 3195 CpG sites that correlated significantly with stool butyrate levels in PD patients (Benjamini–Hochberg FDR *q* < 0.05, robust linear regression) (Fig. 3A) and 2950 CpG sites that were significantly changed in PD relative to controls (Benjamini–Hochberg FDR *q* < 0.05, robust linear regression). In a previous study, we identified genes epigenetically altered in cortical neurons from PD patients.^33^ Genes containing the butyrate‐associated methylated cytosines in blood cells significantly overlapped with those genes altered in PD patients' blood cells (relative to controls) and with those genes altered in PD patients' prefrontal cortex neurons (relative to controls) (genes containing modified cytosines at Benjamini–Hochberg FDR *q* < 0.05, Fisher's exact test *P* < 0.05) (Fig. 3B,C). Pathways involving those genes epigenetically altered in PD or with butyrate were identified and included neurodevelopment, cell development, synaptic transmission, metabolism, and signal transduction (Fig. 3D).

### Epigenetic Links between Butyrate, Bacteria and Leucocytes Differ between Cell Types

**FIG 4:**
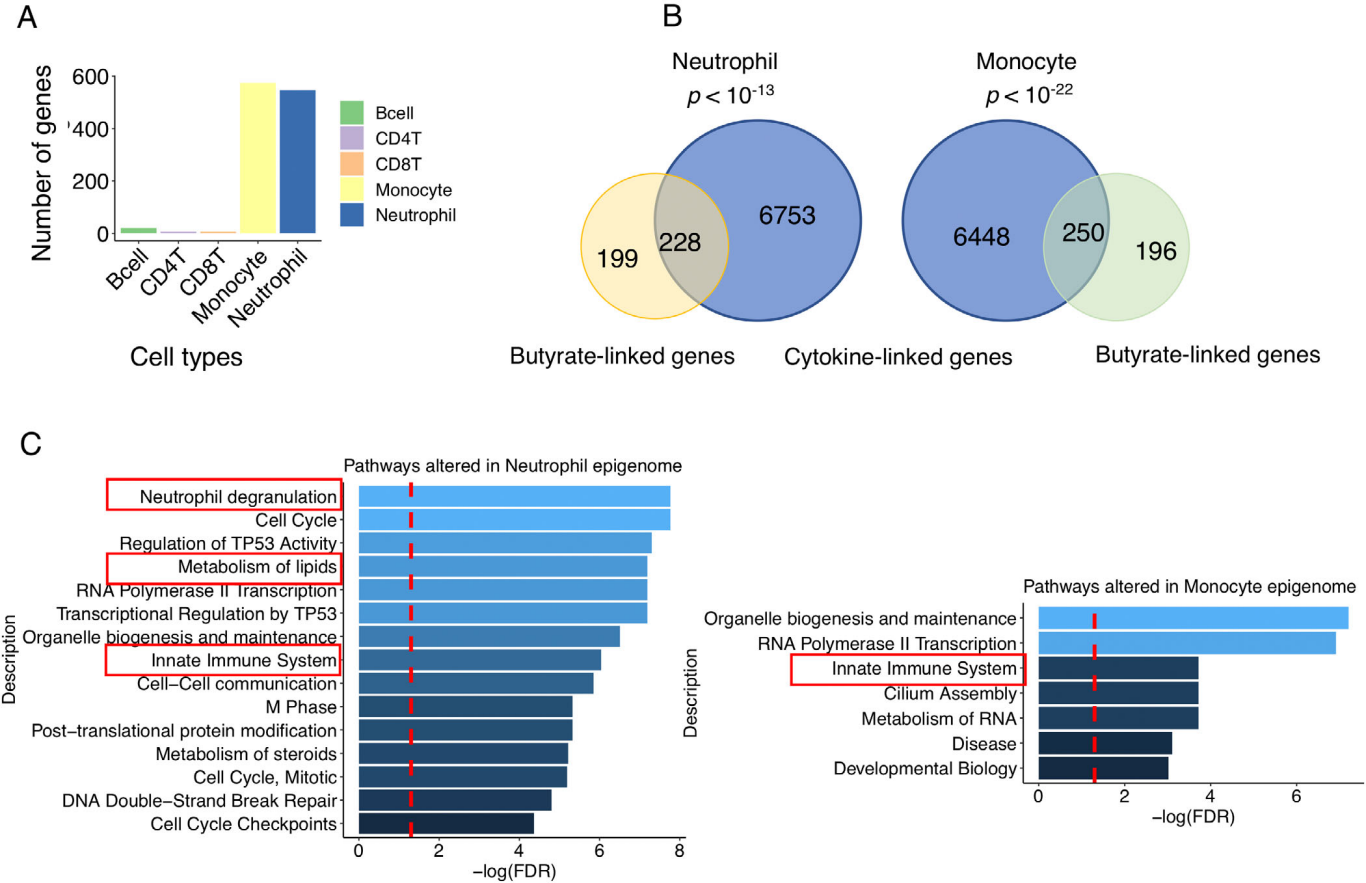
Blood cell types are differentially linked to butyrate. (**A**) Significant methylation sites (related gene numbers) linked to butyrate in the epigenome of neutrophils, monocytes, CD8^+^T cells, CD4^+^T cells, and B cells, respectively. (**B**) Epigenetically altered genes linked to cytokines overlap with those epigenetically linked to butyrate in monocytes and neutrophils. Fisher's exact test, *P* < 0.05. (**C**) Gene set enrichment analysis of the monocyte and neutrophil genes that are epigenetically altered and linked to butyrate. PD polygenic risk pathways, including innate immune systems, metabolism of lipids and neutrophil degranulation, are significantly altered. Significant threshold Benjamini–Hochberg FDR *q* < 0.05. [Color figure can be viewed at wileyonlinelibrary.com]

To test if leucocyte epigenetic changes are associated with fecal butyrate, we used cell‐type‐specific resolution epigenetics, TCA,^38^ to analyze the epigenome for each cell type (Fig. S2). To test the epigenetic links of butyrate on different immune cell types, we analyzed the significant methylation sites linked to butyrate in the epigenome of neutrophils, monocytes, CD8^+^T cells, CD4^+^T cells, and B cells. All cell types were linked differently to butyrate, with neutrophils and monocytes containing the largest number of genes that were epigenetically linked to butyrate (Fig. 4A). Levels of inflammatory stool cytokines were derived from a previously published study of the same samples.^32^ We also analyzed the methylated cytosines that were correlated with blood inflammatory cytokine levels. There was a large overlap between the genes epigenetically linked to butyrate and those epigenetically associated with levels of inflammatory cytokines (TNF, IL6, CXCL8, IL4, IL1B, IL10, IFNg, IL13, IL12p70, IL2, and LBP) in both monocytes and neutrophils (Fisher's exact test, *P* < 0.05) (Fig. 4B). Pathway analysis shows that epigenetic alterations in three recently identified PD polygenic risk pathways^61^ (neutrophil degranulation, metabolism of lipids, and innate immune system) in the monocyte and neutrophil epigenome were linked to butyrate levels (Fig. 4C). We also analyzed the links to other SCFAs, and our results showed that the genes epigenetically altered in neutrophils were most strongly associated with butyrate compared to other SCFAs (Fig. S3a) and that there were no significant PD polygenic risk pathways linked to other SCFAs in neutrophils (Fig. S3b). We further analyzed the significant methylation sites linked to bacterial genera that correlated with butyrate levels and/or depressive symptoms (Table APPENDIX S1). Like butyrate, *Roseburia* and *Romboutsia* were linked mostly to epigenetic alterations in innate immune cells. *Prevotella* and *Deltaproteobacteria_unclassified* showed stronger links to the epigenetic status of T cells. These results indicate that immune cell functions are specifically altered in PD patients and that they might be epigenetically altered by butyrate and gut microbiota.

### Epigenetic–Genetic Correlation between Diseases and Butyrate‐Associated mDNA Regions

**FIG 5:**
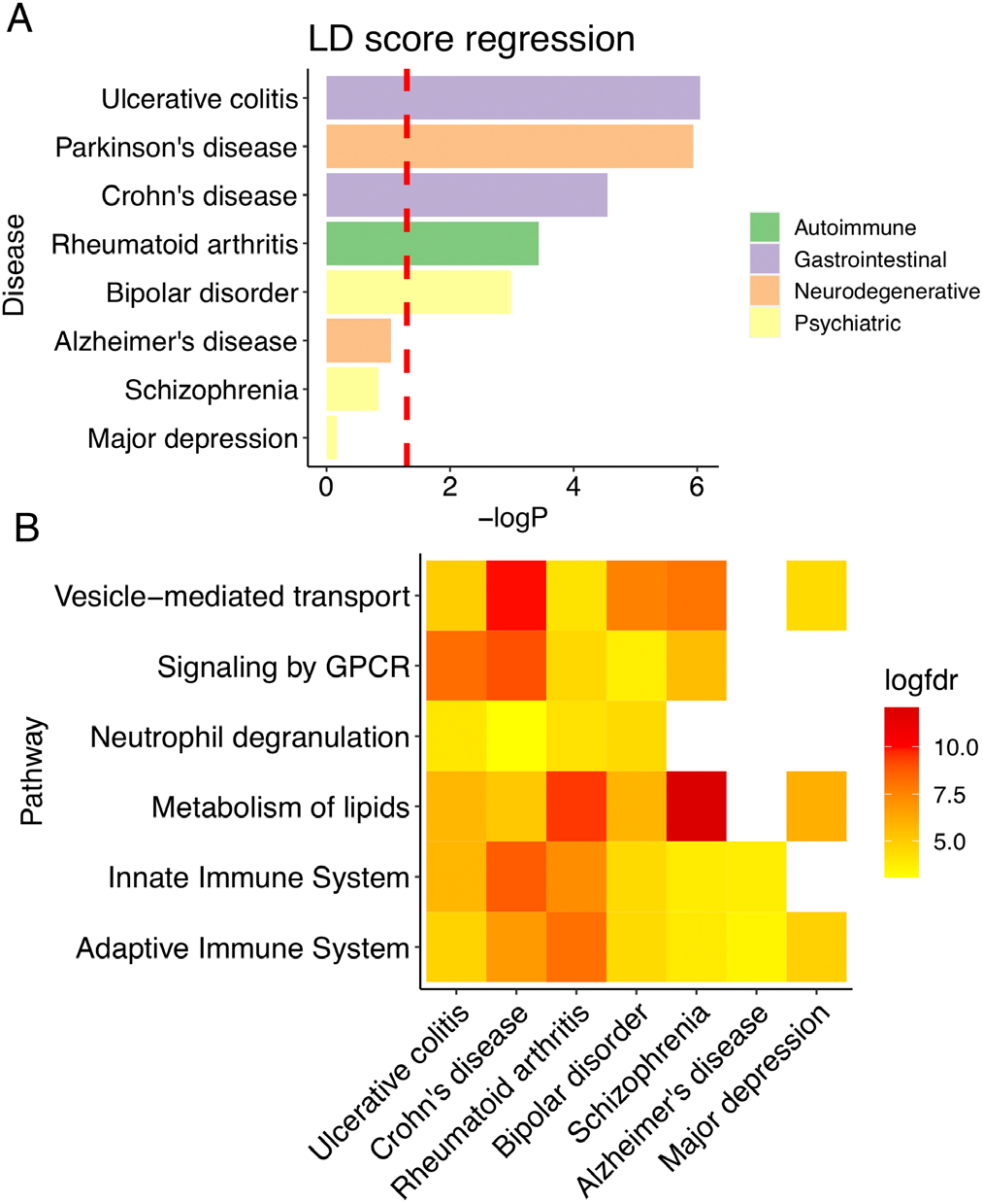
Epigenetic–genetic correlation between butyrate‐associated mDNA (methylated DNA) regions and the GWAS (genome‐wide association studies) of other diseases. (**A**) LD (linkage disequilibrium) score regression was performed between butyrate‐associated mDNA regions and the GWAS summary statistics of Parkinson's disease, ulcerative colitis, Crohn's disease, rheumatoid arthritis, bipolar disorder, Alzheimer's disease, schizophrenia, and major depression, respectively. *P* < 0.05 is used as the significant threshold. (**B**) Gene set enrichment analysis of the location of common genetic regions in Parkinson's disease, butyrate‐associated mDNA regions, and those diseases in panel A. The log(FDR) of six PD polygenetic risk pathways without known PD risk loci was plotted.^61^ Benjamini–Hochberg FDR (false discovery rate) *q* < 0.05 is considered as significant threshold; white squares indicate nonsignificant associations. [Color figure can be viewed at wileyonlinelibrary.com]

To investigate the epigenetic–genetic correlation, we ran a linear regression model to compare the results of methylation analysis to the GWAS of the odds risk ratios for PD and other diseases, including GI (ulcerative colitis, Crohn's disease), autoimmune (rheumatoid arthritis), neurodegenerative (Alzheimer's disease), and psychiatric diseases (bipolar disorder, schizophrenia, and major depression), and our DNA methylation study, adjusting for LD scores. We found that butyrate‐associated mDNA regions are most strongly related to GWAS loci linked to PD and inflammatory bowel diseases. Further significant links were found with the GWAS loci of rheumatoid arthritis and bipolar disorder (linear regression, *P* < 0.05) (Fig. 5A). In contrast, Alzheimer's disease, schizophrenia, or major depression GWAS loci were not linked to butyrate‐associated mDNA regions. Pathway analysis was performed on the common genomic regions (butyrate‐associated mDNA regions, GWAS loci of PD, and each of the other diseases, respectively). All of the six PD polygenic risk pathways without known PD risk loci^61^ were significantly altered in ulcerative colitis, Crohn's disease, rheumatoid arthritis, and bipolar disorder (FDR *q* < 0.05) (Fig. 5B). The top 15 significant pathways are shown in Figure S4. These results indicate that butyrate‐associated epigenetic changes may contribute to observed epidemiologic links between PD and GI, autoimmune, and certain psychiatric diseases.

## Discussion

Several studies have found alterations in microbial composition and metabolites in PD, but the mechanisms linking these changes to PD and its symptoms are not well understood.^8^, ^9^, ^11^ The bacterial metabolite butyrate is a strong HDAC inhibitor, with known epigenetic effects.^62^ Several studies have shown the ability of butyrate to affect the innate and adaptive immune system as well as to alter the blood–brain barrier permeability.^23^, ^27^, ^63^ This study is the first to examine the possible role of epigenetic changes as a link between gut microbiota, its metabolites, and the pathophysiology of neural and immune cells in PD.

In this study, genome‐wide DNA methylation profiling revealed significant epigenetic changes in the leucocytes and neurons of PD patients that overlap with genes, the methylation of which is linked to fecal butyrate levels, including loci in PD risk genes.^33^ Furthermore, butyrate levels correlated most strongly with abundance of bacteria belonging to the genera *Romboutsia* and *Roseburia*, known SCFA producers.

A major finding of our study is that the epigenome of specific immune cell types in PD is differentially linked to fecal butyrate levels and fecal bacterial counts. In particular, neutrophil and monocyte epigenomes show the strongest links to butyrate. We have also shown that the neutrophil epigenome is linked less to other SCFAs. A recent study reported that neutrophil degranulation is potentially linked to PD risk.^61^ Our gene set enrichment analysis suggests that butyrate‐linked epigenetic changes may impact this pathway. Several studies have reported an increase in neutrophils in PD patients, including studies using flow cytometric and epigenetic profiling approaches, which is consistent with our cell deconvolution results from methylation profiling.^64^ However, our study for the first time decomposed the estimated epigenome of each cell type and investigated the epigenetic change from different immune cell types in PD and in connection to SCFAs. Our results suggest that butyrate may impact PD through epigenetic effects on innate immune cells and PD‐related genes.

A limitation of this study is that blood SCFA levels were not available. The exact dynamics that related fecal butyrate levels with blood leucocyte and brain epigenetics are not known. While a significant proportion of microbial‐released butyrate is rapidly taken up and consumed locally in the gut, butyrate can cross the epithelial barrier and enter the circulation via the portal vein.^65^ Microbiota‐derived butyrate impacts histone acetylation in multiple tissues.^66^ Whereas concentrations in the portal vein are still considerable, concentrations in peripheral blood appear to be relatively low.^65^ Thus, leucocytes become exposed to butyrate mostly in the gut wall and portal vein, whereas the impact of butyrate in systemic venous blood can be expected to be less and influenced by liver function. Epigenetic changes in blood leucocytes may impact inflammation systemically and in the brain.^27^ At physiological concentrations, butyrate's impact on brain metabolism and hippocampal neurogenesis has been shown in pigs.^67^ Although our findings support the importance of epigenetic mechanisms, their relative impact on the physiological effects of butyrate in the brain as compared to other mechanisms remains to be established.

Interestingly, our results suggest that patterns of butyrate‐related epigenetic changes in PD are most similar to those found in inflammatory bowel disease and clearly less similar to those found in Alzheimer's disease. Although evidence is mixed for irritable bowel syndrome,^68^ in particular inflammatory bowel diseases^69^ have been related to an increased risk of PD and Alzheimer's disease, but associations are stronger for PD.^70^ Our results suggest that microbiome‐related epigenetic modulation could be a mechanism relating GI disorders and PD. Also, bipolar disorder^71^ has been related to an increased PD risk, and our results support a role for common epigenetic mechanisms in this context. In contrast, we could not find significant overlap with epigenetic patterns found in schizophrenia^72^ and depression,^73^ which points to a lesser impact of epigenetics relating these disorders to PD. Recent meta‐analyses suggest alterations in SCFA‐producing bacteria in several psychiatric disorders and Alzheimer's disease, warranting further research in this context.^74^, ^75^ Interestingly, some overlap was observed with epigenetic patterns of rheumatoid arthritis, which reportedly is associated with a decreased PD risk.^76^, ^77^, ^78^ Rheumatoid arthritis has also been related to the gut microbiome, but changes have been somewhat contrary to those observed in PD, for example, increase in *Prevotella* abundance in arthritis but decrease in PD.^79^

In this study, we partly reanalyzed microbiome and metabolite data with methods not used in the previous publications.^31^, ^32^ We observed that fecal bacterial butyrate is inversely correlated with depressive symptoms (GDS‐15) in PD patients. Although there is no correlation between bacterial butyrate and other nonmotor symptom–related scales that include depressive‐related items (UPDRS I, NMSS, and NMSQ), GDS‐15 is more specific for assessing depressive symptoms. Although we were able to reproduce PD‐related microbiota alterations and identify decreased butyrate levels using earlier methods, we gained important new insights. Fecal butyrate and counts of the genera *Prevotella*, *Romboutsia*, and *Roseburia* were negatively correlated with depressive symptoms in PD patients, potentially implicating bacterial metabolites in this important nonmotor PD symptom.

In sum, combining metabolite, microbiome, clinical data, and DNA methylation profiling, our study is the first to reveal a possible relation between gut microbiome metabolite production and epigenetic changes, implicating immune and neural pathways in PD patients with potential impact on depressive symptoms. Furthermore, our results point to microbiota‐dependent epigenetic modulation as a potential pathway linking inflammatory bowel diseases and PD. Further research on altered bacterial metabolism and its impact on host physiology may reveal new biomarkers and therapeutic targets for PD.

## Author Roles

Conceptualization: A.X., E.E., P.L., J.G., L.L.M., and V.L.; data curation: E.E., V.T.E.A., M.C.H., K.R., L.P., P.A.B.P., M.G.T., P.A., and F.S.; formal analysis: AX; funding acquisition: J.A.P., P.B., L.B., F.S., and V.L.; investigation: P.B., L.B., F.S., and V.L.; methodology: A.X., L.B., and V.L.; visualization: A.X.; writing—original draft: A.X.; writing—review and editing: A.X., E.E., S.G., P.B., L.B., P.A.B.P., V.T.E.A., M.C.H., K.R., M.G.T., and F.S. The authors read and approved the final manuscript.

## Full financial disclosures for the previous 12 months

P.B. has received commercial support as consultant from Axial Therapeutics, Calico, CuraSen, Fujifilm‐Cellular Dynamics Inc., IOS Press Partners, LifeSci Capital LLC, Lundbeck A/S, Idorsia, and Living Cell Technologies Ltd. He has received commercial support for grants/research from Lundbeck A/S and Roche. He has ownership interests in Acousort AB and Axial Therapeutics.

S.G. receives commercial support as consultant from Coleman Research and Biogen.

V.T.E.A., P.A.B.P., L.P., P.A., and F.S. have patents issued (FI127671B, EP3149205B1, and US10139408B2) and pending (US20190137493A1, US20210109098A1, and EP3789501A1) that are assigned to NeuroBiome Ltd.

F.S. is founder and CEO of NeuroInnovation Oy and NeuroBiome Ltd., is a member of the scientific advisory board, and has received consulting fees and stock options from Axial Biotherapeutics. F.S. has received grants from the Academy of Finland, the Hospital District of Helsinki and Uusimaa, OLVI‐Foundation, Konung Gustaf V:s och Drottning Victorias Frimurarestiftelse, the Wilhelm and Else Stockmann Foundation, the Emil Aaltonen Foundation, the Yrjö Jahnsson Foundation, and Renishaw and honoraria from AbbVie, Orion, GE Healthcare, Merck, Teva, Bristol Myers Squibb, Sanofi, and Biogen.

M.G.T. is an ex‐employee of Xencor Inc. and coinventor of dominant‐negative TNF inhibitor (XPro1595) as well as consultant for INmune Bio and holds stock ownership in the company. She is a consultant for and/or collaborates with Longevity Biotech, Cerebral Therapeutics, Innoviva, iMetabolic Pharma, Amylyx, Biogen/IONIS, Nanobiotix, and Jaya. She serves on the Medical Scientific Advisory Board of the Alzheimer's Association and the World Parkinson Coalition and is advisor for the Weston Family Foundation, The Michael J. Fox Foundation for Parkinson's Research, and the Quebec Parkinson's Network. She is editor‐in‐chief of Nature's Parkinson's Disease and serves on the editorial boards of *Science Advances*, *Experimental Neurology*, *Neurobiology of Disease*, *Journal of Neuroinflammation*, *Journal of Parkinson's Disease*, and *PLoS ONE*. Her research is funded by The Michael J. Fox Foundation, the NIH, and the Parkinson's Foundation.

L.B. was supported by the Farmer Family Foundation and the Michigan State University Gibby and Friends versus Parky Parkinson's Disease Research for this project. Other support she received include NIH R01 MH118211 and MJFF grant 010296.

K.R., P.L., J.A.P., A.X., L.L.M., M.C.H., and E.E. have nothing to disclose.

## Supporting information


**APPENDIX S1.** Supporting Information

## Data Availability

Microbiota data are available at the European Nucleotide Archive (accession number PRJEB27564) (https://www.ebi.ac.uk/ena/browser/view/PRJEB27564). Other data and files utilized in this study are available from the corresponding authors upon reasonable request.
