# Supplementary material for: Bacterial Butyrate in Parkinson's Disease Is Linked to Epigenetic Changes and Depressive Symptoms
Source: Mov Disord. 2022 Jun 20;37(8):1644–53. doi: 10.1002/mds.29128 (PMC9545646; doi:10.1002/mds.29128)
Supplement: Supplementary file 1 — APPENDIX S1. Supporting Information [file MDS-37-1644-s001.docx]

Fig. S1:

A: Short chain fatty acids changes in PD patients relative to controls were identified (robust linear regression, adjusting for age, sex, smoking status and BMI). B: Bacterial genera which were altered in the stool from PD patients relative to control group (Benjamini-Hochberg FDR *q* < 0.05, metagenomeSeq ZIG model). C: Left figure: phylogeny of the 71 bacterial genera included in the analysis. Middle figure: correlations between microbes and GDS (adjusting for age, sex, smoking status and BMI, metagenomeSeq ZIG model). Right figure: correlation between butyrate and genera (read count) in PD patients. Left table: bacteria which significantly correlated with GDS (Benjamini-Hochberg FDR *q* < 0.05, metagenomeSeq ZIG model). Right table: bacteria which significantly correlated with butyrate levels (Benjamini-Hochberg FDR *q* < 0.05, Pearson correlation).

Fig S2:

A: Blood cell percentage in PD compared to control. CIBERSORT was used to perform the cell type decomposition from EPIC methylation array. B: Epigenome of different blood cell types was estimated by Tensor Composition Analysis (TCA) based on the M value of methylated cytosines and the cell percentage. C: For the sanity check, we deconvoluted the estimated epigenome using CIBERSORT again. The corresponding cell type dominates in each epigenome, respectively.

Fig S3:

A: Significant methylation sites (related gene numbers) linked to SCFAs in the epigenome of neutrophils, monocytes, CD8^+^T cells, CD4^+^T cells and B cells. B: Gene set enrichment analysis on the neutrophil genes that are epigenetically linked to propionate. Significant threshold Benjamini-Hochberg FDR *q* < 0.05. We found no significant pathways for genes that were epigenetically linked to acetate, isobutyrate, isovalerate and valerate.


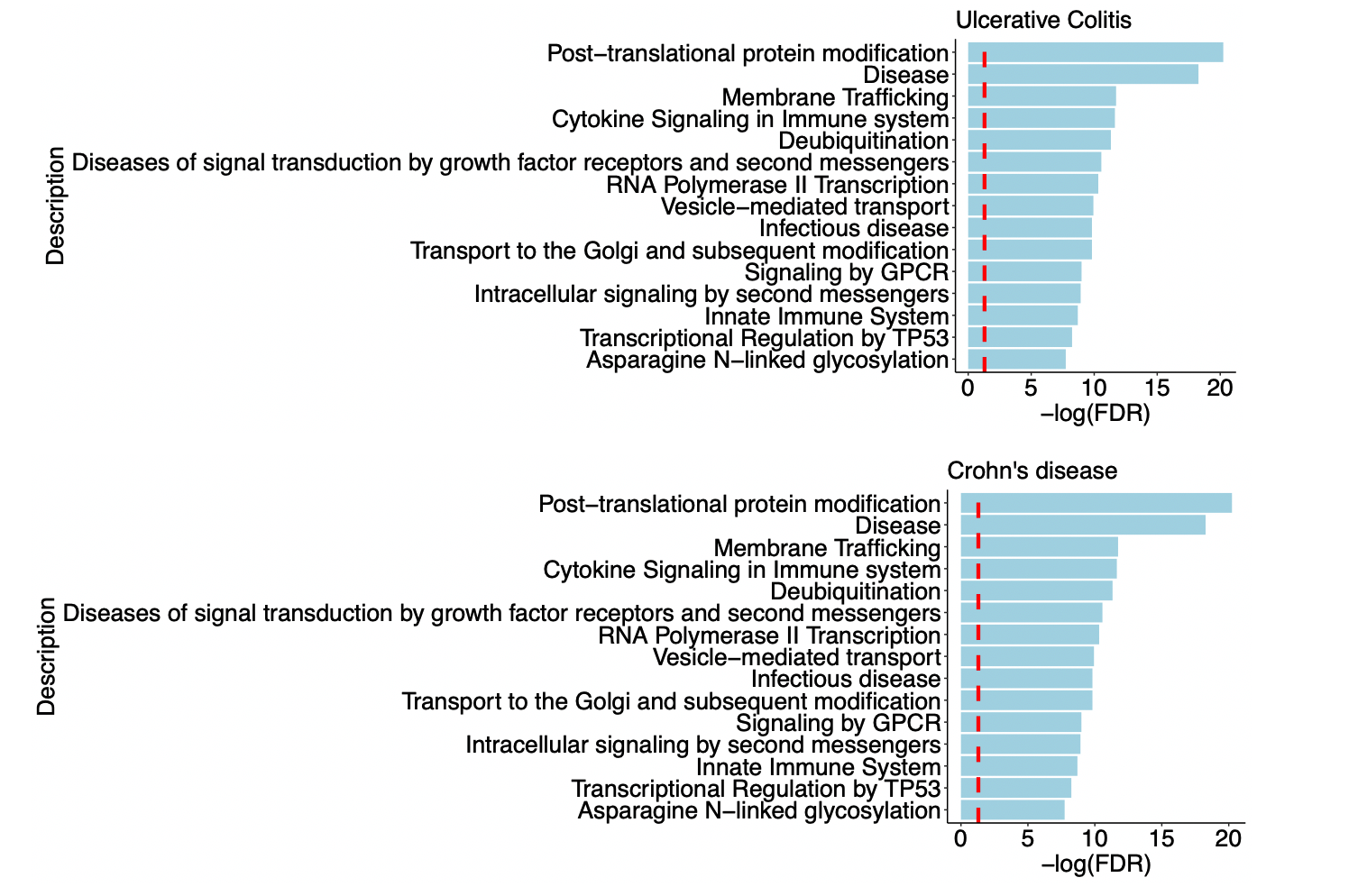

Fig S4: Gene set enrichment analysis on the location of common genetic regions in both Parkinson’s disease, butyrate-associated mDNA regions and ulcerative colitis and Crohn’s disease, respectively. The top 15 significant pathways are shown. Significant threshold Benjamini-Hochberg FDR *q*< 0.05.


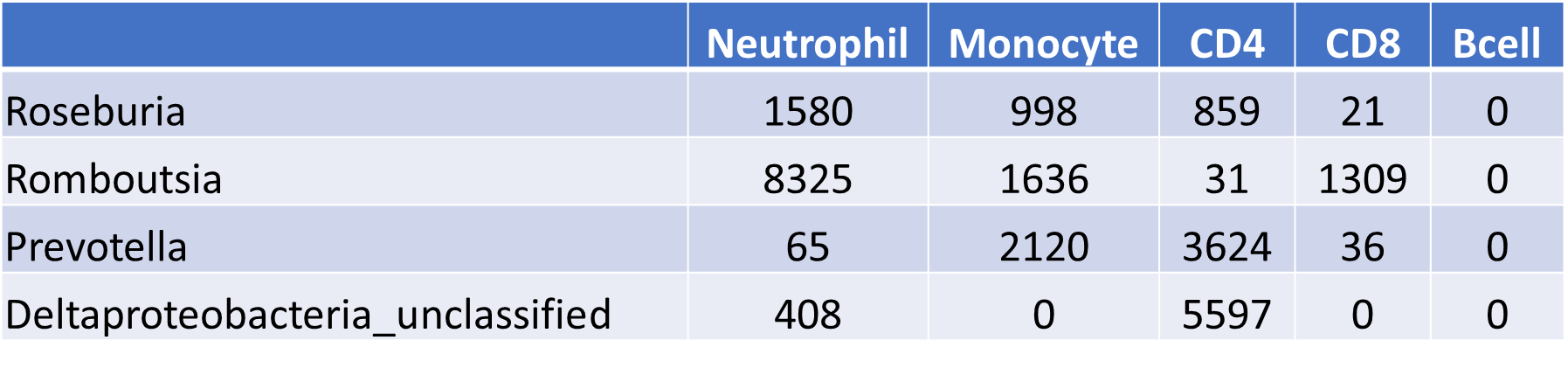


Table S1: Epigenetic alterations in blood cell types are differentially linked to gut bacteria. Significant methylation sites (related gene numbers) linked to bacterial genera in the epigenome of neutrophils, monocytes, CD8^+^T cells, CD4^+^T cells, and B cells, respectively.
